# Coping with ill-health: health care facility, chemist or medicinal plants? Health-seeking behaviour in a Kenyan wetland

**DOI:** 10.1186/s12914-019-0199-1

**Published:** 2019-06-06

**Authors:** Carmen Anthonj, Peter Giovannini, Thomas Kistemann

**Affiliations:** 1 GeoHealth Centre, Institute for Hygiene and Public Health, University Hospital Bonn, Bonn, Germany; 2 Water Institute, Department of Environmental Sciences and Engineering, Gillings School of Global Public Health, University of North Carolina at Chapel Hill, Chapel Hill, USA; 3 Natural Capital and Plant Health Department, Royal Botanic Gardens Kew, London, Ardingly UK

**Keywords:** Cultural context of health, Health behaviour, Health risk perceptions, Pastoralists, Period prevalence, Sustainable livelihoods framework, Traditional medicine

## Abstract

**Background:**

Sub-Saharan African wetlands, settlement areas to growing populations, expose their users to diseases as necessary health infrastructure remains underdeveloped.

**Methods:**

Mixed methods were adopted to assess the health-seeking behaviour of different exposure groups (farmers, pastoralists, service sector workers) in a Kenyan wetland community. Based on a cross-sectional survey (*n* = 400), syndromic surveillance was linked to health-seeking event analysis. In-depth interviews with community members (*n* = 20) and experts (*n* = 8) enabled the integration of healthcare user and provider perspectives.

**Results:**

Health-seeking behaviour in the wetland was determined by physical/infrastructural, natural/environmental, financial/socioeconomic and social/demographic factors, as well as human/cultural aspects such as traditional preferences rooted in health beliefs. Community members had different strategies of coping with ill-health and few symptoms remained untreated. Whether via a health care facility admission, the visit of a chemist, or the intake of pharmaceuticals or medicinal plants: treatment was usually applied either via a healthcare service provider or by the community members themselves.

An undersupply of easy-to-reach healthcare options was detected, and healthcare services were not available and accessible to all. The widely-practiced self-treatment of symptoms, e.g. by use of local medicinal plants, mirrors both potential healthcare gaps and cultural preferences of wetland communities.

**Conclusions:**

Integrated into an overall health-promoting wetland management approach, widely accepted (cultural) realities of health-seeking behaviours could complement health sector service provision and help *ensure healthy lives and promote well-being for all* in wetlands.

## Background

In arid and semiarid parts of the world, many people cope with water scarcity and food insecurity by settling near wetlands. Such ecosystems are being used extensively and increasingly especially in Sub-Saharan Africa (SSA) [1], with adverse implications on the users’ exposure to water-related infectious diseases [2–4]. Wetlands in SSA lack suitable development for exploitation by human beings, with necessary infrastructure in terms of disease prevention - most importantly water, sanitation and waste management services, and personal and environmental hygiene - often unavailable or insufficient [5, 6].

Since potentially prevalent diseases affect quality of life and productivity, consequently affecting overall socioeconomic development, adequate health care options are vital in order to respond to and meet the demand for remediation in such settings. Considering and understanding the behaviour and decision-taking of wetland communities in the case of ill-health can provide valuable information to guide future health interventions and health-promoting wetland management.

Little research has been carried out to determine the level of healthcare utilization among wetland users [7], although it can be assumed that such ecosystems per se are not well equipped and even ‘underserved’ when it comes to human health infrastructure and service provision. Health care facilities might be distant and difficult to access, thus affecting the health-seeking behaviour of those suffering from ill-health. This paper contributes to the knowledge base on health-seeking behaviour in wetlands by adopting a syndromic surveillance approach of self-reported symptoms [6] and related health-seeking event analysis of four different exposure groups in the Kenyan Ewaso Narok Swamp.

### Health-seeking behaviour: a reference to livelihoods

Health-seeking behaviour determines whether or not individuals suffering from diseases receive treatment or cure. Health-seeking behaviour research focuses on the first recognition of symptoms and follows the ill individual through different stages of formulating and handling ill-health - from deciding whether or not to seek care to actual pursuit of health through recovery. Health-seeking behaviour is conceptualized as a ‘sequence of remedial actions’ taken to rectify ‘perceived ill-health’ [8].

The sustainable livelihoods approach [9, 10] can be applied to health-seeking, which is described as *Health Access Livelihood Framework* [11]*,* integrating the five dimensions of access to healthcare services in resource-poor settings [12]. These dimensions include the availability, accessibility, affordability of services, adequacy and acceptability of health services. Access refers to the interaction between the healthcare services, broader policies, institutions, organizations and processed, and livelihood assets individuals can mobilize and combine in a particular vulnerability context. Furthermore, access is determined by cultural norms, subjective preferences and medical traditions with the recognition of illness and treatment seeking depending on individual, community and societal access to livelihood assets [11, 12].

According to this theoretical framework, wetlands (natural capital) contribute both to their inhabitants’ livelihoods, and to the generation of income (financial capital). Social networks provide support (social capital) while infrastructure, roads and means of transport (physical capital) facilitate care-seeking, which is also determined by popular, traditional, and biomedical knowledge (human capital) on diseases and disease transmission providing perspective on how to behave when suffering from ill-health. Multiple healthcare utilization strategies may be adopted depending on severity of ill-health, and access to services and assets [13].

**Fig. 1:**
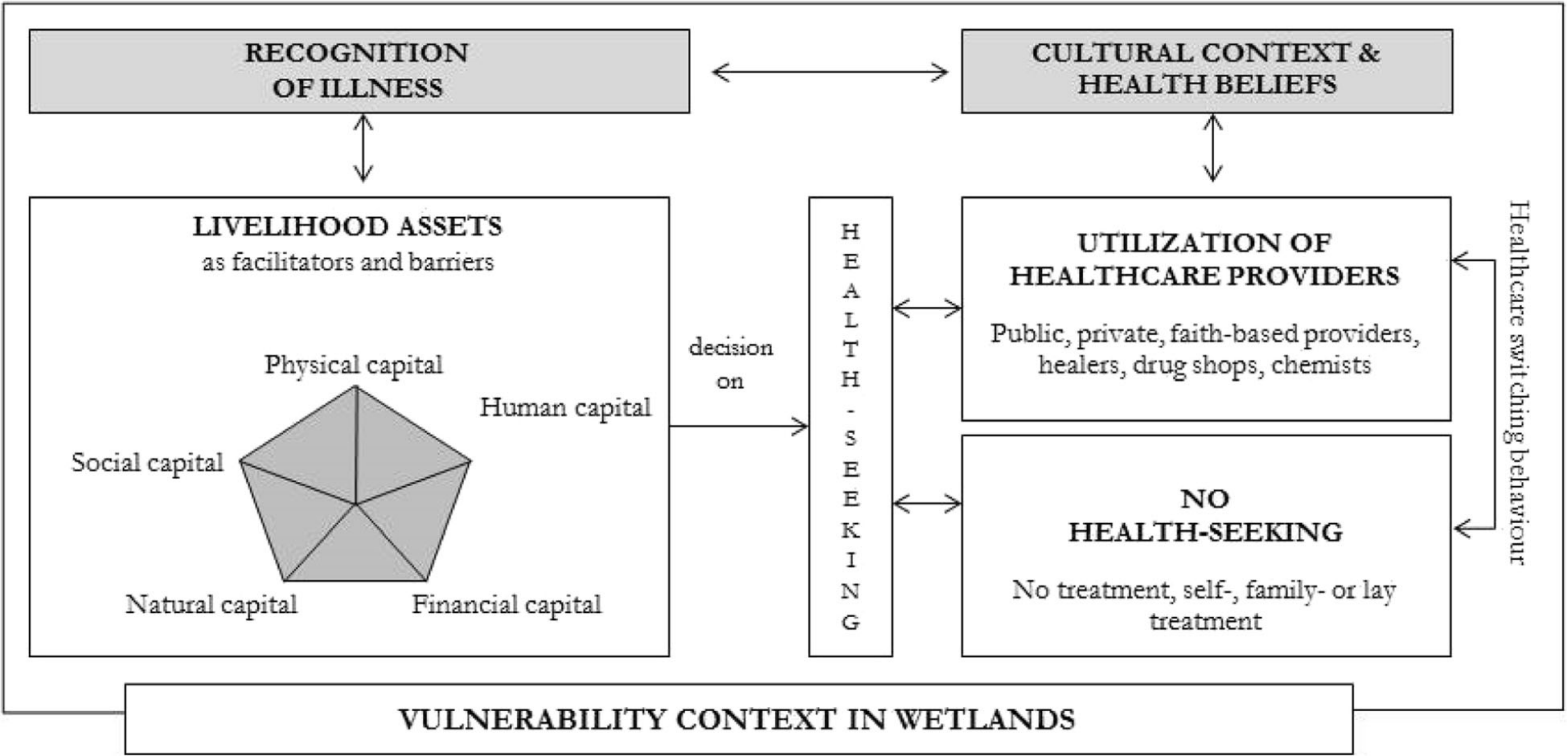
Health-seeking behaviour in a wetland context

These assumptions were integrated into a conceptual framework (Fig. 1) which considers the decision-taking to health-seeking behaviour after recognition of ill-health as mediated by the cultural context and health beliefs, against in in the vulnerability context of wetlands.

### Health and the health sector in Kenya

Kenya is challenged with socioeconomic inequalities, infrastructural deficiencies, and adverse health indicators amidst different geographic settings and different population groups (Table 1), even though health indicators reflect an upward trend [14]. Despite the government’s efforts to tackle environmental health per Vision 2030 and the Sustainable Development Goals (SDG) per a number of programmes, much remains to be done. The Kenyan health system struggles to provide appropriate service delivery to those suffering from ill-health [15], and shortage of health professionals intensifies the tense situation [16, 17].

**Table 1:** Population and health indicators for Kenya

Population	Total population	46,050,414
	Population density (pop/km^2^)	73.9
	Life expectancy at birth [years]	53
	Median age [years]	19.5
	Employment rate [%]	60
	Literacy rate (aged 15 and older) [%]	87
	Poverty gap at $1.90 a day [%]	11.7
	Gini index	48.51
Nutrition	Underweight (weight for age) [%]	11
	Stunted (height for age) [%]	26
Child health	Under-five mortality rate (deaths per 1000 live births)	49
	Infant mortality rate (dying between birth and age 1 per 1000 live births)	36
	Immunization among children (12–23 months) [%]	97
Maternal health	Total fertility rate (children/woman)	3.14
	Maternal mortality rate (per 1000 live births)	0.510
General health	Health expenditures [% of GDP]	5.7
	Total government health funding (per capita) [KES] = USD?	1585
	National Health Insurance Fund (NHIF) coverage [%]	26.7
	Physicians density (per 100,000 population)	0.20

Sources: MoH (2015), WHO (2016), KNBS (2015), KDHS (2014)

The public health sector (national referral hospitals, county and sub-county referral hospitals, health centres, and dispensaries) is complemented by the private sector (private for-profit), non-governmental (NGO), and faith-based organization (FBO) facilities in remote communities. In addition, pharmacies provide health services. The government pursues a sector-wide approach that integrates the combined efforts of all providers in order to achieve ‘health for all’ [15]. There are 0.2 physicians per 1000 Kenyans (vs. 16 in the United States), 0.86 nurses and midwives, 0.05 pharmaceutical workers and 0.1 other health workers. Along with the conventional Western health services, traditional health services provided by healers are used as a source of primary care [18, 19]. The healthcare utilization rate in Kenya is approximately 77% for those who are ill [15].

Particularly in rural areas, where 76% of the population live and depend on natural resources, farming and subsistence activities, the access to health services remains challenging. The situation in wetlands has not yet been assessed, although such ecosystems, in the face of water scarcity, are increasingly important [20], and subject to increasing in-migration.

## Methods

A mixed-method approach was adopted in order to assess the health-seeking behaviour of people reporting ill-health in the Ewaso Narok Swamp. Quantitative data collection was combined with qualitatively assessed local knowledge and perceptions.

### The study area: a wetland in semiarid Kenya

**Fig. 2:**
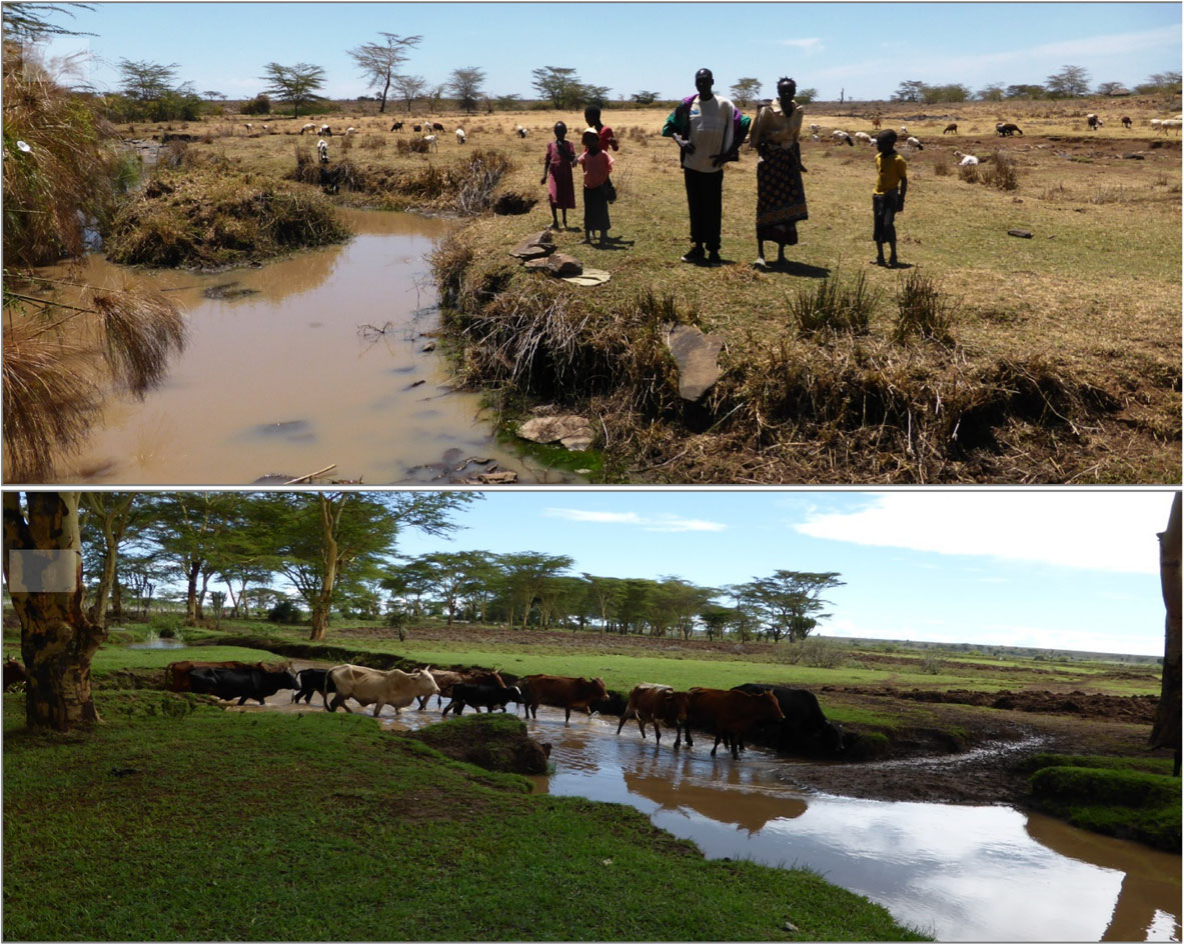
The study area: the Ewaso Narok Swamp in semiarid Kenya

The study was carried out in the Ewaso Narok Swamp, a rural floodplain in Laikipia, Kenya (Fig. 2).

Located by the town and administrative centre Rumuruti, the wetland is fed by the Aberdare Mountains and receives seasonal floodwater from the Eng’are Narok and Mutara Rivers [22]. In an area with seasonal, but low erratic rainfall (two distinct rainy seasons from March to May and in November), the Ewaso Narok Swamp provides an important source of freshwater, livelihood, place of concentrated anthropogenic activities and immense ecological and socioeconomic importance to a growing population [2, 5, 6, 21].

**Fig. 3:**
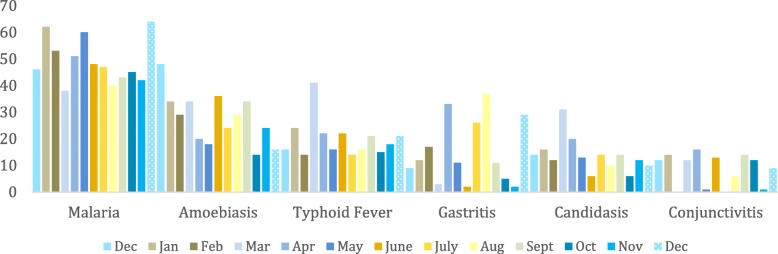
Rumuruti district hospital admission from December 2013 – December 2014. * The different seasons are illustrated in different colours. Blue shades are used for months in the rainy season. Orange and brown shades are used for months in the dry season

The inhabitants of the Ewaso Narok Swamp live in an environment with multiple prevalent diseases: Data from the largest health care facility in the area indicate that malaria, gastrointestinal diseases, typhoid fever and diarrhoeal diseases are currently the main drivers for medical consultation. The admission rates of all diseases show seasonal variation, with peaks of typhoid fever at the beginning (March), and malaria at the end of the rainy season (May) (Fig. 3) [2, 22].

## Data collection

Data were collected from healthcare users and providers (target population, experts) with different methods (cross-sectional survey, in-depth interviews) from January to March 2015 in the Ewaso Narok Swamp (Table 2).

**Table 2:**
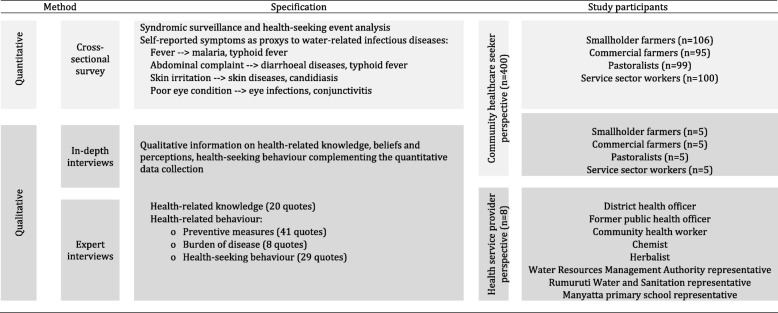
Methodology and data collection on health-seeking behaviour in the Ewaso Narok Swamp

### Cross-sectional survey with syndromic surveillance and health-seeking analysis

A cross-sectional survey was conducted with adult household heads working as smallholder (sh) (*n* = 106) and commercial farmers (co) (*n* = 95), nomadic pastoralists (pa) (*n* = 99) and service sector workers (se) (*n* = 100). The four groups were identified at the study site as a basis for commensurate samples. Smallholder and commercial farmers, as well as service sector workers were randomly sampled. Pastoralists, due to their nomadic lifestyle, were identified based on snowball sampling [2, 5, 6]. The different occupational groups were chosen based on the assumption that different interaction with wetland water exposes them at different extents to different water-related infectious diseases [2, 3, 6, 22], thus initiating different health-seeking behaviours [2]. Whereas commercial farmers were constantly and directly exposed to water during their irrigation activities of horticultures, smallholder farmers interacted with water at a lesser extent, as they mainly grew less water-intensive crops. Pastoralists stayed mainly in the semiarid outskirt areas of the wetland and came only occasionally to the wetland in order to water their livestock. Service sector workers rarely interacted with the wetland. The groups’ household characteristics, socioeconomic status and school education differed [2, 5] (Table 3).

**Table 3:** Characterisation of different user groups in Ewaso Narok Swamp

	Smallholder farmers (*n* = 106)	Commercial farmers (*n* = 95)	Pastoralists (*n* = 99)	Service sector (*n* = 100)
Characteristics				
Household head interviewed (male/female %)	29/71	31/69	49/51	80/20
Number of household members (mean)	5	5	6.5	4
Number of children in household (mean)	2	2	3	1.3
Qualitative description of socio-economic status	medium	medium	low	high
School education of respondents (years)	4	7	2	10
Never attended school (%)	36.8	14.7	63.3	6

Retrospective syndromic surveillance was adopted [2, 6, 23]. The respondents reported symptoms which they had experienced during a reference period of the four weeks preceding the day of the survey (=period prevalence), capturing symptoms including abdominal complaints, fever, and poor eye and skin conditions. The symptoms were used as proxies to infectious disease risks (Table 2) [2, 3, 6]. This syndromic surveillance [23] approach enabled the provision of disease information in an environment where no conventional surveillance could be applied [24]. For each self-reported illness episode, the behaviour was recorded by event analysis [25].

In addition, a survey addressed general information about occupation, household socio-demographics and wetland utilization of household heads. It was conducted orally in English, Kiswahili, Kikuyu, Masai, Samburu, and Turkana by a team consisting of the lead researcher and five trained research assistants from Kenyatta University. Part of this training was piloting the data collection tools and procedures and adjustments to these tools where needed prior to the study. A part of the training was dedicated to the (re-)translation of health-related concepts in the Ewaso Narok Swamp [2, 22].

### In-depth interviews with target population and experts

Of the household survey respondents, key informants for semi-structured, open-ended in-depth interviews were systematically identified by anchor questions. The interviews aimed at capturing knowledge on and explanations for health-seeking behaviour, perceptions, experiences, high-risk groups and setting-specific particularities of the four wetland user groups was represented with five interviewees (*n* = 20 in total) (Table 2) [2, 5, 22].

Experts (*n* = 8) representing the health sector, the water and sanitation sectors, as well as the educational sector [2, 22] served as a supplementary source of information in addition to and for triangulation with the target group [26].

The questionnaire and the guides for in-depth interviews with community members and experts have been developed by the lead author and are published elsewhere [2].

### Secondary data on hospital admissions

The data gathered from the Rumuruti District Hospital on hospital admissions between December 2013 and December 2014 were an important additional source of information (Fig. 3) [2, 22] used for triangulation.

## Data analysis

For the quantitative data analysis, descriptive statistics were calculated and included frequencies for all variables of interest, stratified by user groups, self-reported symptoms, and health-seeking behaviour in order to illustrate varied behaviours. The quantitative data was triangulated with qualitative explanations from community members and experts. The data analysis was guided by the theoretical framework on health-seeking behaviour in wetlands (Fig. 1), informed by the sustainable livelihoods framework and by the access and barriers to healthcare provision theory [2].

The audio-recorded qualitative data were transcribed with the easytranscript® and analysed with ATLAS.ti7® software. Categories were predefined based on the main issues addressed by the respondents [2].

## Results

### Burden of self-reported ill-health

**Fig. 4:**
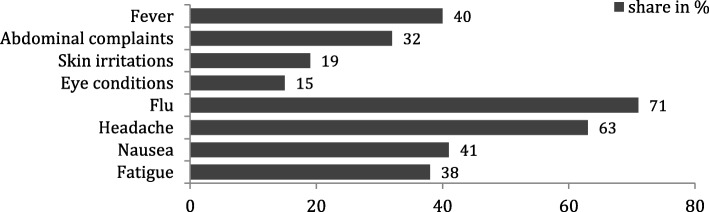
Syndromic surveillance of self-reported symptoms in a wetland community of the Ewaso Narok Swamp (n = 400)

The burden of self-reported ill-health was high in the wetland community: Of all community members interviewed (*n* = 400), 385 reported 1421 symptoms, corresponding to 96% suffering (temporarily) from ill-health during the four-week recall period. Flu (71%) and headache (63%) were reported most. Four symptoms are analyzed in more detail (Fig. 4, Table 4) [2].

**Table 4:** Self-reported symptoms in four week recall period, stratified by user groups

	Smallholder farmers (*n* = 106)		Commercial farmers (*n* = 95)		Pastoralists (*n* = 99)		Service sector workers (*n* = 100)		Total (*n* = 400)	
	n	%	n	%	n	%	n	%	n	%
*Self-reported symptoms*										
Abdominal complaints	39	36.8	31	32.6	27	27.3	32	32.0	129	32.3
Fever	42	39.6	38	40.0	39	39.4	41	41.0	160	40.0
Skin irritation	21	19.8	23	24.2	13	13.1	19	19.0	76	19.0
Poor eye condition	20	18.9	10	10.5	20	20.2	11	11.0	61	15.3

**Fig. 5:**
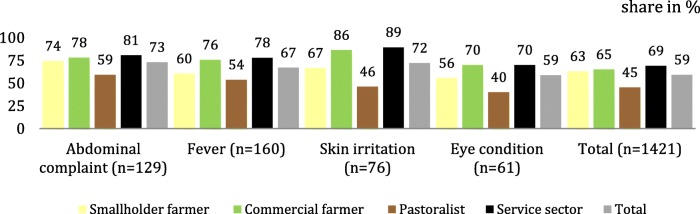
Health-seeking behaviour during last self-reported symptoms in February 2015, stratified by user groups [%] (Fig. 5)

Similar period prevalence of fever was reported by all different groups of wetland users. Differences became apparent in terms of the other symptoms: smallholder farmers predominantly suffered from abdominal complaints (37%), commercial farmers reported skin irritations (24%), and poor eye conditions were mainly experienced by smallholder farmers (19%) and pastoralists (20%). Less abdominal complaints and skin irritations were experienced by pastoralists as compared to farmer and service sector groups (Fig. 5) [2].

### Health-seeking behaviour

Healthcare was sought for 60% (*n* = 851) of symptoms. Health-seeking behaviour varied by symptoms, and was most common for abdominal complaints, skin irritations (74% each), and fever (67%). Only half of those reporting poor eye conditions (52%) sought care providers [2].

The service sector workers made most use of service providers (69%) and the pastoralists sought healthcare least often (45%), regardless of the type of symptoms. Not only the decision whether or not to seek healthcare differed according to the user group, but also the type of service provided. Among respondents who decided to make use of a service provider, a public health care facility (72%) was the most commonly used option (Table 5) [2].

**Table 5:** Health-seeking behaviour, stratified by symptoms and groups [%]

	by symptom					by group				
	Abdominal complaint (*n* = 95)	Fever (*n* = 107)	Skin irritation (*n* = 56)	Eye condition (*n* = 32)	Total (*n* = 290)	Smallholder farmers (263 symp.)	Commercial farmers (211 symp.)	Pastoralists (157 symp.)	Service sector workers (220 symp.)	Total (*n* = 851 symp.)
Public facility	65	79	71	66	72	75	48	66	84	72
Private facility	20	18	16	16	16	15	25	13	10	16
Chemist	11	2	7	13	9	8	11	12	7	9
Faith-based provider	3	2	5	3	3	2	4	8	0	3
Non-governmental provider	1	0	0	3	1	1	0	1	0	1

symp. is usead as an abbreviation and stands for symptom

Choosing a public health care facility was reported to be common during the in-depth interviews, and described as dependent on the severity of ill-health.

*‘Most people go to the hospital when they seek healthcare.’* (sh2).

*‘Depending on the health condition, the people who are sick go to hospitals or clinic.’* (sh4).

**Fig. 6:**
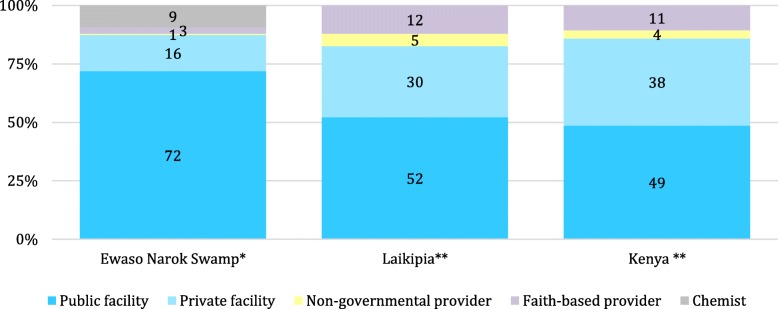
Health-seeking in the wetland compared to county and national level. * Health-seeking during last self-reported symptom in the Ewao Narok Swamp, own data (2015); ** National data on the district Laikipia and on the national level in Kenya, provided by the Ministry of Health (2015)

**Fig. 7:**
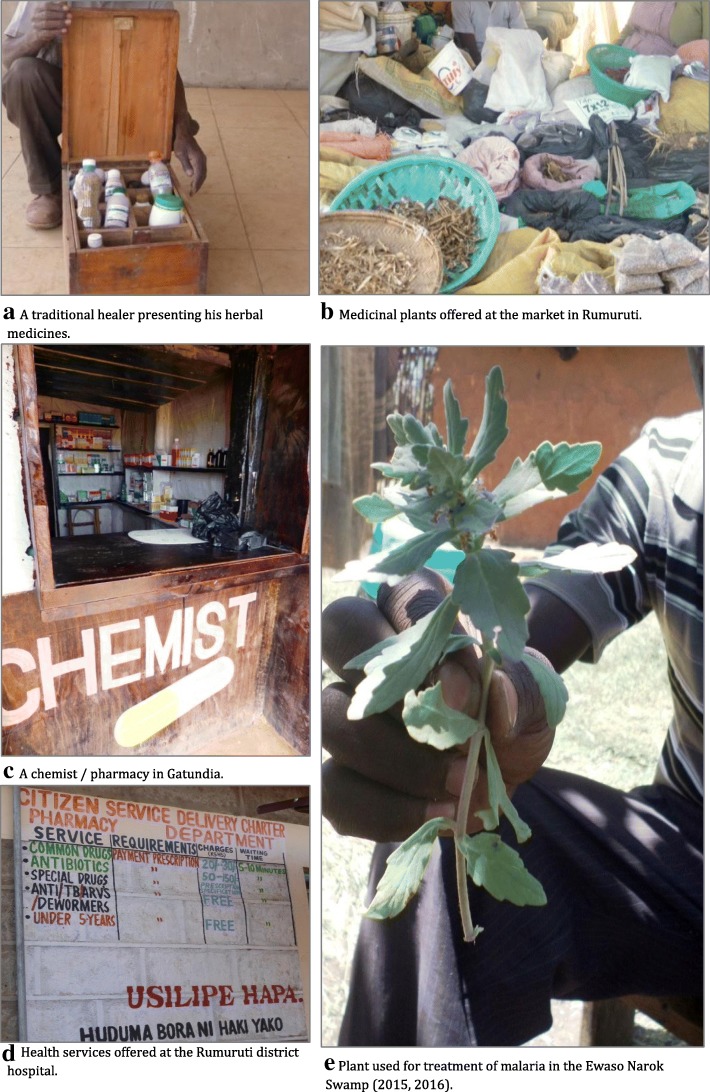
Healthcare options in the Ewaso Narok Swamp

Private health care facilities were sought by 16% of community members, and chemists by 9% (mainly for eye conditions and abdominal complaints) (Fig. 6, Fig. 7). The choice of care provider also varied by group [2].

### Reasons for not seeking healthcare: a community perspective

No need for seeking healthcare or receiving external treatment was perceived by on third of community members reporting to suffering from symptoms. Not seeking care providers in the Ewaso Narok Swamp had multiple causes, most commonly self-treatment (55%). Financial barriers held 8% of community members back from seeking healthcare, and percentages differed according to the self-reported symptoms (Table 6) [2]. Instead of remaining entirely untreated, community members chose self-medication with medicinal plants as an alternative to other, more costly healthcare options:

*‘A big challenge is that the hospitals are so far away.* ( …) *If you don’t have the means for the transport, you have a problem.’* (co4).

*‘Another problem is when we get sick we do not have easy access to the hospital* ( ….). *So we treat the people with medicinal herbs.’* (pa3).

**Table 6:** Reasons for not seeking healthcare, stratified by symptoms and groups [%]

	by symptom					by group			
	Abdominal complaint (*n* = 34)	Fever (*n* = 53)	Skin irritation (*n* = 19)	Eye condition (*n* = 26)	Total (*n* = 561)	Smallholder farmers (*n* = 39)	Commercial farmers (*n* = 22)	Pastoralists (*n* = 48)	Service sector workers (*n* = 20)
Self-treatment with medicine	32.4	28.3	26.3	7.7	26.1	34	34	13	48
Self-treatment with herbs / milk	35.3	37.7	36.8	23.1	29.1	11	8	67	4
No necessity	23.5	28.3	36.8	42.3	36	47	45	12	47
No money	8.8	5.7	0	23.1	7.5	8	11	5	1
Distance to facility	0	0	0	3.8	1.3	0	2	3	0

A substantive share of community members regarded care-seeking as unnecessary for poor eye conditions (42%) and skin irritation (37%). Not seeking care did not necessarily imply foregoing treatment. Instead, community members often treated themselves mainly for fever (75%) and abdominal complaints (68%) [2].

*‘You can cure stomach aches and even malaria and headaches. When you feel you are weak, you help yourself, you get those herbs from the bush.’* (co2).

*‘You just pick the leaves and then you mash them, you crush them, you add some cold water, give a teaspoon in the morning and afternoon and then in the evening.* [You use it] *mostly* [against] *malaria. For abdominal disorders; you find that this one* [showing another plant] *also acts the same.* (…) *I was shown by my grandmother, you see my grandmother was herbalist.’* (co1).

The comparison of different user groups in terms of reasons not to seek care providers revealed that while the farmers had similar reasons not to seek care, service sector workers and pastoralists differed. For the service sector workers, pharmaceutical medicine was the major alternative to seeking healthcare and also played the most important role compared to other groups (48% compared to 34% farmers, 13% pastoralists), while distance to facilities or money were less of a barrier. The group of pastoralists mainly applied herbal medicine and milk for treating symptoms as an alternative to seeking care providers (67% compared to 11% or less among all other groups) [2].

*‘People who like herbal medication are Samburu and Turkana* [pastoral tribes], *the other tribes would rather go to the hospital than use medicinal herbs.’* (co3).

*‘We* [pastoralists] *have medicinal plants and we have livestock so we slaughter one or two and we mix it with herbs and then we take and feel better. We make a soup, or we can use the blood and the milk. The farmers do not know these medicinal plants, sometimes they ask us. Some pastoralists even sell these plants*.’ (pa5).

Some community members perceived the effectiveness of medical attention as ineffective, not sufficient or even useless, which caused shifts from having sought healthcare at health care facilities towards self-treatment of symptoms with herbs:

*‘Even this typhoid that we are getting, even if it is treated* [by healthcare providers] *we don’t get healed, so we take medicinal herbs.’* (sh3).

The pastoralists perceived least self-reported symptoms as requiring treatment (12%) as opposed to the other groups (45%) [2].

### Reasons for not seeking healthcare: a health service provider perspective

The service providers expressed their concerns about the low level of health-seeking at health care facilities in the wetland. A community health worker described the priority setting among people who struggle with financial constraints, which often is to the disadvantage of costly hospital admission.

*‘The people are suffering from diseases but they don’t have any money to go to the hospital and to get treatment. At a facility, you pay 50 bob* [KSH]. *That is for administration. Then after that, it will depend on the disease. Being told to go to the laboratory that is where things become bad, since they are charging very much. So people might not be going to a health facility. It is not because they are not sick. People are sick but they don’t go to a hospital because they don’t have money. They have to set priority. If you need food, you prefer to buy some unga* [maize flour] *and rely on the free traditional medicines from the bush.’* (CHW).

The low coverage of health care facilities was a shortcoming to service utilization in the wetland according to a former Public Health Officer:

*‘The challenge is we don’t have the required health facilities, they are not enough.* ( …) *The problem is the distance because of the time and the cost … ’* (former PHO).

An adverse consequence of not seeking healthcare, highly relevant and challenging to the healthcare system, was formulated by a chemist:

*‘Possibly, they* [the wetland users] *can go unreported because in those swampy areas, mostly they don’t come up to the health centre; not unless they are critically ill. They know it is there but they cannot go to the hospital because they cannot afford, because it is so far and you know, when they do diagnose it’s too late and possibly patients don’t survive.’* (Chemist).

According to the local chemist, not seeking healthcare was common, and she insisted on advising community members to visit health care facilities when suffering from severe conditions:

*‘…*[the community health workers] *are trying to advise and insist them* [ill people] *to visit health facilities, even those whose culture might not allow to do so. Many people are coward and don’t dare to go to the doctors and think they might be charged a lot of money. Now with our advice as mashinani daktari’s* [community doctors] *people are cooperative.’* (CHW).

The complementary traditional medical option used by many of the community members was promoted by a traditional healer claiming that each possible disease, including typhoid, malaria, diarrhoeal diseases could be cured by herbs from the wetland and its surroundings:

*‘Herbs are an alternative medicine. Some people do go to the hospital for a long time without being cured, so they transfer, they seek alternative medicine, just herbs.* ( …) *There are so many diseases that can be cured*. ( …) *The medicine is all around me, there is no need to go to the chemist. The herbal medicines are very good.’* (Herbalist).

He described himself able to use traditional practices, measures, ingredients and procedures to cure diseases. The former Public Health Officer, however, neither believed in the efficiacy of medicinal herbs, nor in the medical knowledge of traditional healers:

*‘Me personally, I don’t trust them.* [The medicinal plants] *won’t work. I don’t believe in somebody who has not been trained in anything to do with disease, who has never seen a class of a medical school. Although some learn from their parents. It is just the traditional belief.’* (former PHO).

## Discussion

Our results indicate a high burden of self-reported symptoms among community members in the investigated semiarid wetland setting, where the vast majority (96%) of interviewed community members reported ill-health during the four-week reference period. This burden of ill-health is much higher than the 11% found in a study from Zambia [27], or the 40% in a Tanzanian wetland [28]. Considering the self-reported symptoms as proxies to diseases such as malaria, typhoid fever, diarrhoeal diseases, eye and skin diseases potentially present in wetlands [2, 3, 6], makes information on health-seeking behaviour a vital foundation, based on which health care providers may develop health interventions.

### Preference of care: health care facility, chemist or medicinal plants

Healthcare services were sought for 60% of self-reported symptoms in the investigated wetland. Healthcare services utilization varied by symptom: more community members sought a care provider for abdominal complaints and skin irritations (74%) and fever (67%) than for poor eye conditions (52%). These figures are much lower than the 77% of the Kenyan population seeking healthcare in times of ill-health according to Turin [15]. Seeking care from public health care facilities was the most common therapeutic choice and more prominent than private facilities or chemists. Such health-seeking patterns correspond to evidence on health behaviour of pastoralists suffering from malaria in the Kenyan Baringo district [28]. A comparison of health-seeking behaviour in the Ewaso Narok Swamp (Fig. 6) with the county-wide and national data on utilization of healthcare [17] shows a higher share of people using public health care facilities in our investigated wetland, and fewer using private facilities, faith-based providers and non-governmental providers. Most people who sought healthcare made use of the District Hospital, the best equipped facility with most qualified health staff around the wetland, as other clinics existed, but were small and not providing the same quality of services [2].

Ten percent of community members sought healthcare from chemists - a statistic which is included neither in the county- nor in the nationwide healthcare information. Where only few health care facilities are available, chemists play an important role in closing a supply gap, as was also found in Nigeria [29]. It should be noted that chemists provide medicines used for self-medication, so people may make use of chemists to buy medicines rather than for consultation, or alternatively for both [2].

### Determinants of health-seeking behaviour

Our results suggest that the utilization of healthcare services in the study area is determined by numerous factors: access, socioeconomic status, cultural context and health beliefs, perceived severity of ill-health and perceived efficacy of the treatment [2]. The same determinants of healthcare seeking behaviour also were found by Giovannini and Heinrich in a rural Mexican context [30].

Besides existence and accessibility, road infrastructure and distance, including the time, transport and costs involved in travelling determined health-seeking. This supports findings from other contexts [11, 31]. Infrastructure inadequacies and distance were perceived as barriers to access to healthcare by more healthcare providers than community members. This bias reflects the disconnect of perceptions between the service providers and the community seeking care, and indicates how important it is to consider different stakeholders’ perceptions in order to draw a complete health service picture.

Socioeconomic status influenced health-seeking behaviour as well. The use of a health care facility incurs costs for admission, laboratory testing, drugs, hospitalization or accommodation, and alimentation, both for the patients and their company. Possession of health insurance is rare, and even if community members possess such, it covers only a part of the total expenses. Out of pocket payment is common, which transforms the decision to seek a care provider a privilege for those who are able to pay [2, 28, 32].

### Health-seeking as indicator for perceived severity of ill-health

These obstacles turn the health-seeking decision process into a cost-benefit analysis [27], and an indicator for the perceived severity and/or duration of ill-health [33, 34]. Households consider incurring the cost of healthcare only the symptom is perceived as “pressing enough”. Medical knowledge, awareness of diseases and health risk perceptions are major determinants of seeking care [22, 28, 31, 35], and some symptoms are simply perceived as ‘not for hospital’ [2, 36].

### Different health-seeking behaviours among different wetland user groups

The community members working in the service sector visited healthcare providers most, pastoralists least. Pastoral homesteads are usually located in remote terrains, marginalized and distant from tarmac roads and health care facilities [29], which is often a reason against seeking such options of care. Besides living in hard-to-reach areas, pastoralists are culturally and linguistically diverse, and have little access to community health education or broadcasts, while also generally having lower levels of formal education (Table 6) and biomedical knowledge [2, 22, 29, 34]. Pastoralists typically closely adhere to their tradition, culture and habits, have health beliefs, perceptions, understandings and responses to ill-health that differ from other groups [2, 22, 37, 38]. For pastoralists, it is important to approach ill-health through herbalists or traditional healers, embedded within belief system and able to explain the ‘meaning’ of disease [22, 39]. In contrast, service sector workers face the fewest challenges, as they typically live in the town centre - near health care facilities and chemists – have higher socioeconomic status, formal education levels and access to health-related information. These factors may explain their more frequent care-seeking behaviour [2].

### The role of self-treatment and the use of medicinal plants

Self-treatment played an extraordinary role in terms of coping with ill-health in the Ewaso Narok Swamp, as was also found elsewhere [11, 28, 38, 40]. In rural Kenya, as many as 83% of individuals suffering from malaria applied self-treatment as their first choice of care [34]. Although the level of self-treatment was lower in our study area, self-treatment was used for each of the self-reported symptoms. More than half of the community members used medicinal plants, milk, or pharmaceuticals themselves instead of seeking a care provider. The pastoralists stood out, as two thirds of them used local traditional medicine to cure their symptoms. They claimed knowledge on how to prepare local remedies for each specific symptom and disease, including malaria, diarrhoeal diseases and typhoid fever [2]. The same diseases were previously found to be treated with traditional medicines in Uganda [41] and Kenya [29].

In the Ewaso Narok Swamp, extensive local knowledge on the traditional treatment of health conditions existed [2, 22]. ‘Human’ (local knowledge) and ‘natural’ (the wetland) capital (Fig. 1) were highly valued and appreciated by the wetland community, with medicinal plants playing a major role in terms of treatment of ill-health. As the in-depth interviews revealed, the pastoralists in particular would rather use herbs - with knowledge taught by ancestors and handed down through generations - than trust conventional medicine [2]. These findings dovetail with previous research underlining the importance of herbal medicines in Kenya and in other countries in Africa [38, 41].

### Perceived quality of healthcare provision and switching in health-seeking behaviour

Our data confirm that the perceived quality of healthcare provision determines health-seeking behaviour [35]. Moreover, those coping with ill-health applied multiple therapeutic options towards cure: where self-treatment and use of herbs or pharmaceuticals were ineffective, the patients sought care from a health care facility or medical practitioner. Those who initially sought care from formal healthcare providers switched to traditional medicine if foregoing treatment was ineffective. Such decisions were driven by the perceived severity of self-reported symptoms [2]. This switching in health-seeking behaviour in our semiarid wetland setting confirms evidence from Kenya among pastoralists [29] and others [34, 40] suffering from malaria where in order to recover as fast as possible and keep expenditures as minimal as possible, different treatment transitions are undertaken.

### Methodological discussions and limitations

This study aimed at addressing and understanding health-seeking behaviour in the Ewaso Narok Swamp. A cross-sectional study design using syndromic surveillance and event analysis was adopted. The study relied entirely on self-reporting, thus potentially entailing a recall bias - over- and underreporting by the community members, both in terms of symptoms and action taken in response [6]. The recall period was four weeks in the dry season, a time span within which symptoms might have been forgotten or added, thus confounding the results. However, four weeks are not expected to bias the results substantially [2, 6, 42].

Due to the small sample size, only percentages were computed. Therefore, these findings are not per se generalizable beyond the regions studied. However, we triangulated our quantitative and qualitative results with the official health sector data to verify the results. Similar trends to the ones we uncover in our paper may be visible in similar settings.

Although the design and training of this study involved the (re-)translation of health-related concepts beyond biomedical terminology in the multi-cultural context of the Ewaso Narok Swamp through the support of research assistants of different ethnic affiliations, it is likely that understandings of symptoms and ill-health were lost in translation [2, 22].

The cross-sectional design of the survey could only capture the health-seeking behaviour at one point of time and thus not fully account for behaviours in different seasons and years, for which a longitudinal design may have been more useful. It is important to note that the rainy season and flooding can pose different (enhanced) water-related health risks and disease exposures [2, 22] that lead to different health-seeking behaviours (Fig. 3) and at the same time substantially limited accessibility of health services [43], as compared to the dry season, particularly in a semiarid wetland setting [2, 6, 22].

Moreover, considering potential disease risks in wetlands [2, 3, 6, 22], one must keep in mind the co-occurrence of symptoms for one disease (e.g. abdominal complaints and fever both indicating typhoid fever), for which the care-seeking was recorded separately.

## Conclusion

In the Ewaso Narok Swamp, health-seeking behaviour is determined by numerous physical/infrastructural, natural/environmental, financial/socioeconomic, social/demographic, as well as human/cultural factors. Community members have different coping strategies towards ill-health, and few symptoms remain untreated. Whether it is a health care facility, chemist or medicinal plants: community member take action and apply some kind of treatment, either via a healthcare service provider or by the community members themselves. Healthcare services were scarce and community members as well as care providers reported an undersupply of easy-to-reach options, hinting at healthcare services not being available and accessible to all. The widely-practiced self-treatment of symptoms might mirror such weaknesses of healthcare provision [2, 38].

The population around the Ewaso Narok Swamp has been growing in the past 30 years. In a semiarid area, this water resource attracts increasing in-migration of users and this, considering the disease exposure in the wetland, creates an increasing demand for healthcare services, which service providers can hardly keep pace with. Local options and the potential of self-treatment and traditional medicine therefore become ever more important to reach a wider portion of the community [2].

As most disease cases in Sub-Saharan wetlands occur in marginalized communities, distant from effective diagnostic and treatment facilities [22, 44], the use and effectiveness of traditional medicine available on-site should be further validated [2, 40, 41]. The private retail sector could also be strengthened as self-treatment is usually the very first response to an ill-health [2, 11, 34].

Primary healthcare, community health workers and traditional healers become increasingly important in the engagement with underserved and hard-to-reach populations such as pastoralists, and in helping to close the service provision gap in wetlands as found in rural semiarid Kenya [22]. All of these actors need to be considered in a health-promoting wetland management that is adapted to the health needs and realities of local communities and that helps achieve the United Nations Sustainable Development Goal 3 to ‘*ensure healthy lives and promoting well-being for all’*.

## Abbreviations

CHW: Community Health Worker
co: Commercial farmers
DFID: Department for International Development
DHO: District Health Officer
FBO: Faith-based Organization
IHME: Institute for Health Metrics and Evaluations, Kenya
KNBS: Kenya National Bureau of Statistics
KU: Kenyatta University
MoH: Ministry of Health, Kenya
NGO: Non-Governmental Organization
pa: Pastoralists
PHO: Public Health Officer
SDG: Sustainable Development Goal
se: People working in the service sector
sh: Smallholder farmers
SSA: Sub-Saharan Africa
WHO: World Health Organization
